# Current Knowledge on Selenium Biofortification to Improve the Nutraceutical Profile of Food: A Comprehensive Review

**DOI:** 10.1021/acs.jafc.0c00172

**Published:** 2020-03-17

**Authors:** Roberto D’Amato, Luca Regni, Beatrice Falcinelli, Simona Mattioli, Paolo Benincasa, Alessandro Dal Bosco, Pablo Pacheco, Primo Proietti, Elisabetta Troni, Claudio Santi, Daniela Businelli

**Affiliations:** † Department of Agricultural, Food and Environmental Sciences, University of Perugia, Perugia 06123, Italy; ‡ Department of Pharmaceutical Sciences, University of Perugia, Perugia 06123, Italy; § Instituto de Química de San Luis, INQUISAL, Centro Científico-Tecnológico de San Luis (CCT-San Luis), Consejo Nacional de Investigaciones Científicas − Universidad Nacional de San Luis, Chacabuco y Pedernera, Ciudad de San Luis 5700, Argentina

**Keywords:** speciation, micronutrient, metabolite, vegetable, fruit, meat

## Abstract

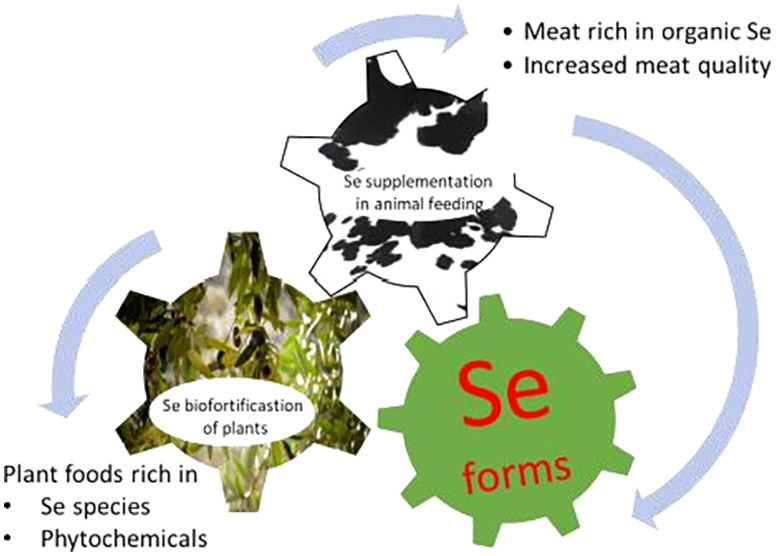

Selenium (Se) is an important micronutrient for living organisms, since it is involved in several physiological and metabolic processes. Se intake in humans is often low and very seldom excessive, and its bioavailability depends also on its chemical form, with organic Se as the most available after ingestion. The main dietary source of Se for humans is represented by plants, since many species are able to metabolize and accumulate organic Se in edible parts to be consumed directly (leaves, flowers, fruits, seeds, and sprouts) or after processing (oil, wine, etc.). Countless studies have recently investigated the Se biofortification of plants to produce Se-enriched foods and elicit the production of secondary metabolites, which may benefit human health when incorporated into the diet. Moreover, feeding animals Se-rich diets may provide Se-enriched meat. This work reviews the most recent literature on the nutraceutical profile of Se-enriched foods from plant and animal sources.

## Introduction

Selenium (Se) is an essential micronutrient, and an adequate intake of this essential trace element is thought to be beneficial for maintaining human health.^1^ It is present in several natural kingdoms, humans, animals, cyanobacteria,^2^ and some plants; it contributes to the control of water status of plants,^3^ prevents oxidative stress, delays senescence, and promotes growth.^4,5^

More than 25 selenium-containing proteins have been identified in mammals and are distributed in different tissues and cells,^6^ having in all cases a role in the regulation of redox processes. Glutathione peroxidase (GPx) is the most studied and well characterized selenoprotein, and its involvement in the detoxification of reactive oxygen species (ROS) has been clearly demonstrated. Similar activity was reported for thioredoxin reductase (TrxR) and selenoprotein P, whereas the analogues K, M, N, and H have a number of different roles in the maintenance of the redox homeostasis of living systems, and iodothyronine deiodinases (DIO) have a fundamental role in the activation of the thyroid hormones.^7^ All these proteins have as a common characteristic the presence of a selenocysteine 21st amino acid in which the catalytic core is a selenol/selenolate stabilized by a amino acidic triad.^8^ Included in the biological processes that can be modulated by Se are not only the cellular response to oxidative stress but also the cellular differentiation, function (including enterocytes and adipocytes), immune response; the redox signaling and protein folding; and the regulation of insulin action and secretion.^9^

People living in the United States and Canada normally have no problems connected with Se deficiency;^10^ on the contrary, those who live in China, New Zealand, and parts of Europe and Russia occasionally show an insufficient intake of this micronutrient due to low levels of Se in soil and, as a consequence, in food.^11^

Se concentration in mammals’ serum ranges between 7 and 14 μg/dL,^12^ and Se is taken in by food as both inorganic forms (such as selenite, SeO_3_^2–^, and selenate, SeO_4_^2–^) and/or organic derivatives (such as the amino acid selenomethionine (SeMet) and selenocysteine (SeCys)). As for many nutrients, several studies in humans have provided evidence of a U-shaped relationship between Se concentration in the blood and the risk of disease, with possible harm occurring both below and above the physiological range for optimal activity of some or all selenoproteins.^13^ High serum Se levels are associated with increased risk such as in the case of diabetes mellitus,^14^ while Se deficiency occurs when the intake is lower than 20 μg/day, and this condition has been correlated to a number of pathologies including cancers, Alzheimer's or Parkinson's disease, male infertility, and thyroidal dysfunctions.^7^

Some plants, in the presence of high levels of inorganic Se, can metabolize and accumulate Se in the form of organic derivatives. This process is important for the plant because it reduces the toxicity of the chalcogen, and at the same time, when bioaccumulation occurs in edible tissues, this process allows the production of Se-enriched foods that have use as a potential nutraceutical for humans and animals.^15^ Moreover, Se biofortification may elicit the production of secondary metabolites, which may benefit human health when assumed with the diet.^16−18^

Therefore, biofortification strategies applied to produce Se-enriched foods could help overcome Se deficiency and its implications in human health and improve the nutraceutical value of food. Despite several scientific works that have dealt with Se-biofortification strategies, the production of Se-enriched foods suitable for animal and human consumption is still challenging.

This review is focused on the Se biofortification of plants to obtain both Se- and phytochemical-enriched foods and feeds, which are potentially useful in increasing, directly or indirectly (i.e., by transfer to livestock meat obtained with Se-enriched feeds), human intake of Se and bioactive compounds. Studies concerning Se content in mushrooms are not included here since the wide literature devoted to this subject would deserve a specific review, taking into account also Se-containing proteins and polysaccharides that are of interest in cancer chemoprevention.^19,20^

Since different Se forms have different bioavailability as well as different metabolic pathways, Se speciation analysis is examined first as a powerful tool to evaluate the Se species in the Se-enriched foods.

## Advances in Speciation Analysis

Total Se concentration (TSeC) in biofortification is determined to evaluate the biofortification efficiency. However, this information is incomplete considering that different Se species possess different bioavailability for humans. It is well-known that organic Se forms (e.g., Se amino acids) are more bioavailable than inorganic forms, such as selenite and selenate; indeed, the human body absorbs more than 90% of SeMet but only about 50% of Se from selenite.^21^

In humans, Se absorption from products of plant origin is much easier than Se absorption from products of animal origin. Therefore, scientists are mostly interested in analyzing Se speciation in plant-derived fortified foods.^22^

The analysis of Se species requires considerations from the treatment of samples to the identification and quantification of these species. The selenol group (−SeH) of SeCys and other Se-amino acids have very low oxidation potential. During extraction procedures, the addition of dithiothreitol (DTT) is advised to avoid oxidation.^23^ Direct analysis of Se species in samples can also be performed by using X-ray absorption near edge structure (XANES) and extended X-ray absorption fine structure (EXAFS).^24^ Similarly, laser ablation (LA) coupled to inductively coupled plasma mass spectrometry (ICP MS) has been used for bioimaging the Se distribution and localization in tissues.^25^

The principal analytical approach to Se speciation has been based on the fractionation and separation of extracts by chromatography (or electrophoresis) while specifically monitoring Se by ICP MS. High performance liquid chromatography (HPLC) has almost universal applicability, and it is the most versatile separation technique, which benefits from a wide array of stationary phases providing different separation modes.^26^

ICP MS can be used for the quantification of Se species, owing to its high sensitivity and element-specific analytical response, independent of the molecular structure, even in case of unidentified Se species. At first sight, it seems there is a full compatibility between HPLC and the traditional sample introduction system of ICP MS, as HPLC provides a typical flow rate in a range of 0.2–1.0 mL min^–1^, which perfectly matches the flow rate range of the traditional nebulizers used (in combination with a spray chamber) for sample introduction in ICP MS. Three different ICP MS sample introduction systems (i.e., a micro concentric nebulizer mounted onto a cyclonic spray chamber, a direct injection nebulizer (DIN), and an ultrasonic nebulizer) were compared in the context of HPLC ICP MS analysis of Se species. The micro-concentric nebulizer combined with a cyclonic spray chamber was found to be the optimal sample introduction system, taking the chromatographic peak shape, sensitivity, and limits of detection (LODs) into account. Ar-based spectral interferences, while monitoring the ion signals of the ^78^Se, ^80^Se, and ^82^Se isotopes, can be solved with methane as a reaction gas in the dynamic reaction cell (DRC) used in ICP MS to eliminate the on-mass.^27^ The quantification accuracy of Se species can be increased by isotopic dilution mass spectrometry (IDMS). The principle of IDMS is based on the alteration of the isotopic ratio of the analyte’s two or more isotopes, by spiking the sample with an isotopically enriched standard. By applying relevant mathematical equations for IDMS and measuring the altered isotopic ratio, the concentration in the sample can be obtained. IDMS can be performed as a species-specific or a species-unspecific analysis.

The identification of Se metabolites can usually be achieved by using traditional techniques, MS and Nuclear Magnetic Resonance (NMR). Electrospray ionization (ESI) in MS is often used either in tandem with ICP MS or as a complementary detector. ESI is a soft ionization mode that can preserve the molecular form of biomolecules, and since its implementation into analytical methods, this instrument has proven to be invaluable for the structural elucidation of molecular species. On the other hand, ESI MS also enables fragmentation of selected molecules, and produced fragments are very often crucial in the identification of unknown molecular species. The identification of novel Se species has been exclusively done by ESI MS, with high molecular mass precision, when high resolution instruments such as Orbitrap, ESI, time of flight (TOF) MS, or ESI MS/MS are used.^25^ In addition, the growing sensitivity of ICP MS detection, owing to collision cell and triple quadrupole mass spectrometers, has resulted in an increasing number of unidentified peaks in HPLC and ICP MS chromatograms.

On the level of selenoproteins, bioinformatics approaches have allowed the putative description of selenoproteomes (sets of Se-containing proteins with genetically introduced selenocystein via a SeCys element). In parallel, the increasing robustness of ESI sources and the advent of high-resolution high-mass-accuracy mass analyzers (notably TOF and Orbitrap) coupled with HPLC continuously increased the number of identified compounds.^26^

## Selenium Biofortification Strategies in Plants

Agronomic Se biofortification has many advantages over direct Se supplementation, since inorganic Se absorbed by the plant is transformed into organic forms, which have a higher bioavailability. Many variables are involved in Se biofortification strategies, such as the Se administration mode (soil fertilization, foliar spray, or hydroponics), Se dose, species and fertilizer form, crop species, and variety and growth stage, to name a few. Indeed, Se species distribution in soils shows that, after irrigation, selenate can be considered as an easily available short-term pool of Se for plants. The long-term pool of Se in the topsoil mainly consists of selenite and organic Se species. These species are readily retained but still sufficiently mobile to be taken up by plants. The formation of elemental Se can be considered as a nonavailable Se pool and is thus the major cause of Se immobilization and long-term enrichment of Se in soils.^28^ In this sense, two years of selenite fertigation in maize (*Zea mays* L.) increased the content of inorganic and organic Se forms,^18^ while irrigation did not affect Se concentration. In rice, selenite uptake promoted organic Se accumulation, but this was mainly stored in roots, a nonedible part of the plant. On the contrary, selenate uptake resulted in the accumulation of selenate in the higher part of the shoots, which is an essential requirement for Se to be transported to the grain.^29^ Foliar application is a valid alternative for Se enrichment of agricultural products.^30^ Compared to Se fertilization to the soil, foliar application by-passes any interference due to soil chemistry and microbiology issues, ensuring a higher efficacy even with low volumes of Se solution. Foliar application of selenite or selenate has been successfully performed to increase the Se content in many crops.^30,31^ Furthermore, the technique paves the road toward the enrichment of plants by costly stable isotopes, which are useful tools in plant physiology research.

In hydroponic systems, as it may be the case in the production of soil-less vegetables and microscale vegetables, Se can be supplied to the water or the nutrient solution.^32,33^

As far as the plant growth stage is concerned, Se may be applied all at once or repeatedly and from sowing to stem elongation, with different outcomes in terms of Se accumulation and partitioning among plant organs.^34,35^ At the vegetative stage, root application of selenomethylselenocysteine (SeMeSeCys) caused the highest water extractable Se content in leaves with major a contribution from organic Se species such as Se amino acid and non-amino acid organic Se. Further investigation at the reproductive stage revealed that foliar application of selenite resulted in the highest total Se content in rice seeds, which was largely attributed to inorganic Se. In contrast, the root application of selenite led to the maximum accumulation of organic Se compounds, which are the most beneficial to human health.^36^ The application of Se during the booting stage resulted in the highest concentration of Se in brown rice due to the highest upward translocation of Se. More than 90% of Se in brown rice was accounted for by organic species, mainly SeMet. The proportion of SeMet in the brown rice decreased with the delay in application time.^37^ In potatoes, foliar application of selenite during the tuber bulking stage was appropriate for the production of Se-rich potatoes.^34^ In broccoli, Se fortification at developmental stages increased SeMeSeCys content.^38^

Finally, the environmental factors (soil characteristics, rainfall, and temperature regimes, etc.) and the cultivation practices (sowing date, fertilization and irrigation schedules, use of growth stimulators, etc.) may greatly affect the Se uptake and partitioning among plant organs. Moreover, both environmental stresses and Se may interfere in affecting the content of secondary metabolites in plant tissues.

For all the aforementioned reasons, reviewing the literature available on Se-biofortified foods is not easy, and any effort to regroup treatments and effects may give arbitrary interpretations that may be questionable. In light of this, the last 10 years of literature is summarized in Tables 1–11, regrouping plant foods by crop types (arable crops, vegetables, microscale vegetables, and fruit trees) and pointing out, for any reference, the plant species and cultivar; the Se source, dose, and application mode; and the main effects of Se biofortification in terms of total and organic Se content and other nutritional traits (such as bioactive compounds and antioxidant activity). Only literature dealing with the content of Se species in edible portions of plants is considered here, neglecting references focused on the effect of Se on plant physiology, biochemistry, and molecular biology. Finally, Table 12 summarizes literature on Se-enriched meat from livestock fed with Se-enriched feed. Since cooking methods could imply losses of Se species, the results reported in the following Tables 1 – 12 are referred to as raw products. Indeed, it has been estimated that around 13.5, 24.0, 3.1, and 46.9% of SeMet were lost during the processes of steaming, boiling, frying, and milking, respectively, while SeCys and SeMeSeCys were completely lost from boiled cereals.^39^

**Table 1:** Cereals: Crop Species, Se Treatment (Se Source, Dose, and Application Mode), and Effects on Total (TSeC) and Organic Se Content and Other Nutritional Traits

species	Se source	dose	type of treatment	TSeC	organic Se	other nutritional traits	references
*Oryza sativa* L. (cv. Xiushui 134)	soil culture: sodium selenite	790 μg of Se pot^–1^ foliar application: 100 μM Se	root treatment foliar application	in rice seeds: Se inorganic > Se organic (foliar application), ↑selenite (root application)	↑Se Amino acid, ↑Non-amino acid organic Se	↑antioxidant capacity; ↑amino acids; ↑Ca, Mg, Zn, Mn	(36)
*Oryza sativa* L. (cv. Premium N° 59, Teyou 59)	sodium selenite sodium selenate	20 g of Se Ha^–1^ (sodium selenite)	foliar application	in grain samples (μg of Se g^–1^): 0.471–0.640	NA^a^	↑Se concentration	(121)
		20 g of Se Ha^–1^ (sodium selenate)					
*Zea mays* L.	sodium selenate	5.0–20.0 g of Se Ha^–1^	field experiment	in grain (mg of Se kg^–1^ DW^b^): 0.042–0.068 (soil application), 0.157–0.306 (foliar application)	NA	↑Se concentration	(122)
			soil application				
			foliar application				
*Zea mays* L. (Dekalb DKC4316, FAO 300)	sodium selenite	200 g of Se Ha^–1^ at low (LH) and high irrigation (HH)	field experiment (soil application; years, 2016 and 2017)	in grain (μg of Se kg^–1^ DW): 1310 (LH) and 1390 (HH), in 2016	SeMet	↑inorganic and organic Se forms, ↑xanthophyll, ↑salicylic acid, ↓hydroxycinnamic acid content, ↑antioxidant activities	(18)
				80 (LH) and 200 (HH), in 2017	SeCys		
*Zea mays* L. (cv. Zhengdan 958)	sodium selenite	Se sprayed and then incorporated (SA): 150–600 g of Se Ha^–1^. Foliar addition (FA): 11–285 g of Se Ha^–1^	field experiment	in grain (μg of Se kg^–1^ DW): 0.6–206.0 (SA), 7.0–2312.0 (FA)	NA	soil and foliar Se: ≈N, P, K, Ca, Mg, Fe, Mn, Cu, Zn contents; ↑Se content	(123)
			soil addition: SA and FA				
*Triticum aestivum* L. (cv. Shannong 1 (purple), Shannong 031244 (blue), and Shannong 129 (white))	sodium selenite	37.50–112.50 g of Se Ha^–1^	field experiment (foliar addition)	in grain (mg of Se kg^–1^ DW): 0.23–0.54	NA	↑Se concentration, ↑gliadin, ↑glutenin, ↓albumin, ↓globulin, ↑iron, zinc, ↓copper, ↓manganese, ↑amino acids, ↑anthocyanins	(124)
*Triticum aestivum* L. (var. BRS 264)	sodium selenate	12–120 g of Se Ha^–1^	field experiment (foliar addition)	in grain (mg of Se kg^–1^ DW): 2.86 (average value at the highest dose)	NA	↑starch content, ↑total soluble sugars, ↑reducing sugars, ↑sucrose, ↑N and antioxidant metabolism	(125)
				in leaves (mg of Se kg^–1^ DW): 1.20–2.32			
*Triticum aestivum* L. (cv. Jordão, bread wheat, TA)	sodium selenate (ate)	4, 20, and 100 g of Se Ha^–1^	field experiment (soil treatment, ST; foliar spray, FS)	in grain (mg of Se kg^–1^ DW): from 0.76 (TA, ate, ST) to 2.98 (TD, ate, FS)	↑SeMet		(200)
*Triticum durum* Desf. (cv. Marialva, TD)	sodium selenite (ite)						
*Triticum durum* Desf.	sodium selenate	10–40 g of Se Ha^–1^	field experiment (foliar spray)	in grain (μg of Se kg^–1^ DW): 457.0–1543.0	↑SeMet	↑Se content	(126)
	sodium selenite						
*Hordeum vulgare* L. (spp. distichum)	sodium selenate	10–40 g of Se Ha^–1^	field experiment (foliar spray)	in grain: (μg of Se kg^–1^ DW) 55–33 and 10–6 for each g ha^–1^ of Se	NA	↑Se concentrations	(127)
	sodium selenite						

a NA: not analyzed.

b DW: dry weight.

## Se-Biofortified Plant Foods

### Arable Crops

Tables 1 and 2 report total and organic Se contents and effects on other nutritional traits of cereal and legume grains, as affected by biofortification strategies. From the results in Table 1, it can be drawn that the fortifying methods used in literature to enrich the crops (foliar spray and soil application) are able to supply the grain with doses of Se suitable for human nutrition; in particular, for rice, the higher Se concentration in grain was achieved by absorbing Se from roots in the form of selenite, while for all the other plant species, the most efficient method of fortification was foliar spray. The nutritional benefits that cereal grain may obtain with Se fortification were an increase in antioxidant activity; a nutrient content higher than in the control; and an increase in amino acids, phenols, anthocyanins, sugars, and Se organic forms. This seems to encourage further research on the possible use of Se-fortified cereals in the diet.

**Table 2:** Legumes: Crop Species, Se Treatment (Se Source, Dose and Application Mode), and Effects on Total (TSeC) and Organic Se Content and Other Nutritional Traits

species	Se source	dose	type of treatment	TSeC	organic Se	other nutritional traits	reference
*Phaseolus vulgaris* L.	sodium selenate	5.0–20.0 g of Se Ha^–1^	field experiment	in grain (mg of Se kg^–1^ DW^a^): 0.05–0.235 (soil application), 0.23–1.24 (foliar application)	NA^b^	↑Se concentration	(122)
			soil application				
			foliar application				
*Lens culinaris* Medikus (subs. Culinaris, cv. PBA Herald XT, PBA Bolt, PBA Ace)	potassium selenate	40.0 g of Se Ha^–1^	field experiment (foliar spray)	in seeds (μg of Se kg^–1^ DW): 201–3327	NA	↑Se concentration	(128)
*Cicer arietinum* L. (cv. Vulcano)	sodium selenate	10.0–40.0 g of Se Ha^–1^	field experiment (foliar spray)	in grain (μg of Se kg^–1^ DW): 714 (selenite), 2721 (selenate) on average	↑SeMet	↑Se content	(129)
	sodium selenite						
*Glycine max* L.	sodium selenite	0.9 mg of Se kg^–1^ of soil	pot experiment (soil substrate)	in bean (mg of Se kg^–1^ DW): 75	↑SeMet		(130)
					↑SeCys		

a DW: dry weight.

b NA: not analyzed.

Table 2 summarizes recent literature on Se biofortification in legumes (bean, lentil, chickpea, and soybean). The results obtained for legumes do not yet make completely clear the nutritional benefits of Se fortification. Both selenite and selenate, as well as both foliar spray and soil addition, are effective in increasing Se content in seeds. Unfortunately, information about the increase in the nutritional quality of Se-enriched seeds is still lacking; however, the ascertained presence of SeMet in chickpea and soybean seeds encourages further research to deepen these studies.

### Vegetable Crops

Much research was also conducted on the Se fortification of lettuce and other leafy vegetables, such as spinach, basil, endive, and chicory. The results are reported in Tables 3 and 4.

**Table 3:** Lettuce: Plant Genotype, Se Treatment (Se Source, Dose, and Application Mode), and Effects on Total (TSeC) and Organic Se Content and Other Nutritional Traits

species	Se source	dose	type of treatment	TSeC	organic Se	other nutritional traits	references
*Lactuca sativa* L. (cv. Susana, Hungary)	sodium biselenite	50–100 ppm Se	field trials	in leaves (μg of Se kg^–1^) 46–1708	NA^a^	↑chlorophyll content	(131)
			soil application			↑catalase (CAT)	
			foliar application			↑ascorbate peroxidase (APX) activities	
			soil:foliar application by ratio 1:1				
*Lactuca sativa* L. (cv. Venezaroxa)	sodium selenite	0–40 μM Se L^–1^	in hydroponics	in leaves (μg of Se g^–1^ DW^b^): selenite, 23.2–50.8; selenate, 57.4–602.0	NA	↑Se concentration	(41)
	sodium selenate						
*Lactuca sativa* L. (var. Romana)	sodium selenate	1–50 mg of Se kg^–1^ of peat.	in pots (some plants grown and Se-fortified in pots were transplanted in open field)	in edible organs, open field experiments: (μg of Se kg^–1^ DW): 21.4–61.3 (in 2012); 24.1–45.5 (in 2013)	NA	↑Se in edible organs	(45)
*Lactuca sativa* L. (var. Capitata, cv. Batavia Rubia Munguía, cv. Maravilla de Verano)	sodium selenite, organic seleno compound, SeCH_3_ organic form some plants were also mycorrhized	40 μg of Se plant^–1^ Se added to the substrate	in pots (greenhouse experiment)	in plants (pg): 439–4501	NA	↑mineral composition, ↑soluble proteins, ↑concentration of nonstructural sugars in shoots	(48)
*Lactuca sativa* L. (var. Capitata)	sodium selenite	0.0–30 μM sodium selenite	in hydroponics	in shoots (mg of Se kg^–1^ DW): selenite,3.7–30.6; selenate, 4.7–43.3	NA	↑Se concentration	(43)
	sodium selenate	0.2–60 μM sodium selenate					
*Lactuca sativa* L. (cv. Vera)	sodium selenite	0–64 μmol of Se L^–1^ with the nutrient solution both as selenite and selanate	in pots (greenhouse experiment)	in shoots (mg of Se kg^–1^ DW): selenite, 0–12; selenate, 0–23	NA	↑Se concentration	(44)
	sodium selenate						
*Lactuca sativa* L. (cv. Philipus)	sodium selenite	5–120 μmol of Se L^–1^ with the nutrient solution both as selenite and selenate	in pots (greenhouse experiment)	NA	NA		(47)
	sodium selenate						
*Lactuca sativa* L. (cv. Philipus)	sodium selenite	5–120 μmol of Se L^–1^ with the nutrient solution both as selenate and selenite	in pots (greenhouse experiment)	in leaves (mg of Se kg^–1^ DW): selenite, 2–38; selenate, 1.5–42	NA	↑Se content	(49)
	sodium selenate						
*Lactuca sativa* L. (cv. Philipus)	sodium selenite	5–120 μmol L^–1^ with the nutrient solution both as selenate and selenite	in pots (green house experiment)	in leaves (mg of Se kg^–1^ DW): selenite, 2.5–40.0; selenate, 1.0–44.0	Cys (mg g^–1^ FW^c^): selenite, 0.48–0.98; selenate, 1.34–2.17. Amino acids (mg of Gly g^–1^ FW): selenite, 0.56–1.07; selenate, 0.50–0.77. Proteins (mg of Alb g^–1^ FW): selenite, 2.30–3.96; selenate, 2.78–2.84	↑Se concentration ↑*O*-acetylserine (thiol)lyase and serine-acetyltransferase activity, ↑Cys concentration	(46)
	sodium selenate						
*Lactuca sativa* L.	sodium selenite	selenite: 1.5 and 5.0 mg of Se kg^–1^ of soil.	in pots (application to a soil substrate, greenhouse experiment)	selenite (mg of Se kg^–1^ DW) in shoots: 0.74–1.11	NA	↑Se concentration	(99)
	sodium selenate	selenate: 1.5 mg of Se kg^–1^ of soil.					
	carboxy methylcellulose (CMC)			selenate (mg of Se kg^–1^ DW) in shoots: 6.21–6.68			

a NA: not analyzed.

b DW: dry weight.

c FW: fresh weight.

**Table 4:** Other Leafy Vegetables: Crop Species, Se Treatment (Se Source, Dose, and Application Mode), and Effects on Total (TSeC) and Organic Se Content and Other Nutritional Traits

species	Se source	dose	type of treatment	total Se concentrations	Se organic	other nutritional traits	references
*Cichorium endivia* L. (var. crispum Hegi)	sodium selenate	0–8.0 μmol of Se L^–1^	in hydroponics (fertigation or foliar spray)	in shoots (mg kg^–1^ DW^a^): fertigation, 1.94–17.61; foliar spray, 0.95–12.67	NA^b^	↑ascorbic acid and total phenolics	(132)
*Cichorium intybus* L. (cv. Anivip and Monivip)	sodium selenate	10 mg of Se L^–1^ (moistening the roots)	in aeroponic system (greenhouse)	in leaves (mg kg^–1^ DW): 139–370 in Anivip cv., 205–460 in Monivip cv.	NA	↑Se content	(133)
*Ocimum basilicum* L. (cv. Tigullio)	sodium selenate	0.5–4.0 mg of Se L^–1^	in hydroponics (floating system)	in stems (mg kg^–1^ DW): 4–21 (1st experiment), 0.98–1.25 (2nd experiment). In leaves (mg kg^–1^ DW): 11–32 (1st experiment), 2–5 (2nd experiment)	NA	↑Se concentration, ↑rosmarinic acid content	(134)
*Ocimum basilicum* L. (var. Red Rubin and Dark Green)	sodium selenate	25 mg of Se m^–2^	foliar applied	in leaves (mg kg^–1^ DW): 2.31–7.01 in Red Rubin var. (first harvest), 1.71–4.08 in Dark green var. (1st harvest)	NA	↑Se content	(55)
*Ocimum basilicum* L. (var. Dark green and Red Opal)	sodium selenate	25 mg of Se m^–2^	foliar applied	NA	NA	↑antioxidant activity, ↑total polyphenol content	(135)
		50 mg of Se m^–2^					
*Ocimum basilicum* L.	not reported	0–120 mg of Se L^–1^	pot experiment (foliar application)	not reported	NA	↓Chlb, ↑Car, ↑antioxidant activity, ↑soluble phenol, ↑proline content	(52)
*Ocimum basilicum* L.	sodium selenate	1–50 mg of Se L^–1^	pot experiment (foliar application)	in shoots (mg kg^–1^ DW): 0.95–150	NA	↑anthocyanin and phenolics, ↓MDA decreased, ≈pigments, ↑total Se content	(53)
*Spinacia oleracea* L.	sodium selenate	0–5.2 μM	in floating system	in leaves (μg kg^–1^ DW): 9.3–15.5	NA	↑Se content, ↓sugars, ≈sucrose, ≈nitrate content	(50)
*Spinacia oleracea* L. (cv. Missouri)	sodium selenite	1–10 mg of Se L^–1^	in hydroponics	in shoots (mg g^–1^ DW): 1.71–3.89	NA	↑micronutrient, ↑antioxidant capacity	(51)

a DW: dry weight.

b NA: not analyzed.

The total Se concentration in the leaves of Se-treated lettuce changed greatly, depending on the Se fertilizer (selenite or selenate) and the method of Se-fortification used (Table 3). The most important benefits due to Se fortification were a decreased nitrate content; an elevated lettuce quality and yield;^40−43^ an increased leaf area, dry weight, pigment content, and antioxidant enzyme activity;^42−44^ a slightly higher shelf life with respect to the control;^45^ an enhanced N and/or S metabolism or total sugar content;^46−48^ and an increased stress tolerance.^49^ As far as lettuce is concerned, the risk of reaching total Se concentrations in the leaves that is too high for the human diet seems to be excessive compared to the little evident nutritional benefits.

For spinach, the only total Se concentration values suitable for human nutrition were those reported by Ferrarese et al.,^50^ who found concentrations in the leaves ranging from 9.3 to 15.5 μg of Se kg^–1^ DW (Table 4). The only benefit of Se fortification shown in these works was an increase of the antioxidant capacity, and actually, an increase of growth parameters has been found to occur only with Se doses^51^ too high to be used for products suitable for human consumption. The studies on basil showed that the benefits due to Se fortification included an enhancement of carotenoids, soluble phenols, proline, and anthocyanin,^52,53^ whereas contrasting effects on biomass increase have been highlighted.^53,54^ The essential oil content was not influenced by Se fortification.^55^ The nutritional benefits obtained from the biofortification of basil have been achieved with doses of Se too high to be compatible with human nutrition. However, this plant material, which is rich in carotenoids, soluble phenols, proline, and anthocyanin, could be used by mixing it with similar untreated plant material to obtain a Se content suitable for human diet.^52,53^ The studies on chicory evidenced an increase in plant yield and antioxidant compounds, such as ascorbic acid and total phenolics.

Particularly relevant are the studies on the Se biofortification of Brassicaceae (Table 5), as these leafy vegetables are Se-hyperaccumulating plants.

**Table 5:** Brassicaceae: Crop Species, Se Treatment (Se Source, Dose, and Application Mode), and Effects on Total (TSeC) and Organic Se Content and Other Nutritional Traits

species	Se source	dose	type of treatment	total Se concentrations	Se organic	other nutritional traits	references
*Raphanus sativus* L. (cv. Saxa)	sodium selenate	5–20 mg of Se plant^–1^ (pot experiment)	pot experiment (soil substrate, foliar application) in hydroponics	pot experiment (μg plant^–1^): in roots, 6.87–15.38 in hydroponics (μg); in roots, 0.007–6.56	pot experiment (mg 100 mg^–1^ tissue FW^a^): ↑SeMetSeCys in roots, 1.62–3.34. In hydroponics (mg 100 mg^–1^ tissue FW): in roots, 0.75–1.51	↑phenolic, ↑cysteine, ↑glutathione, ↑glucoraphanin, ↑total N, ↓polyphenols in hydroponics,: ↑biomass cysteine in root, ↓glutathione both in roots and leaves, ≈polyphenols	(136)
		0.4–1.6 mg of Se plant^–1^ (hydr. experiment)					
*Brassica oleracea* L. (var. Marathon)	shoots of Se-accumulator plant *Stanleyapinnata* L. (powdered plant material, 700 μg of Se g^–1^ DW)	17.5–140 mg of Se lisimeter^–1^	field-installed lisimeters filled with amended soil	in florets (μg of Se g^–1^ DW^b^): 0.5–3.5	in florets (%). (real time SAX-HPLC-ICPMS): 58 SeMet, 15 CysSeSeCys, 7.4 MeSeCys, 6 selenate, 3.1 selenite. (XANES): 55 SeMet and MeSeCys, 23 CysSeSeCys, 18 SeOMet, 4 selenate	↑Se content	(56)
*Brassica oleracea* L. (Var. Heraklion, Marathon, Parthenon, and Naxos)	sodium selenate	25–50 g of Se Ha^–1^	field experiment (foliar application)	in heads (mg of Se kg^–1^ DW): 0.335–1.01	selenate, SeCys_2_, Se-MetSeCys, SeMet, and 2 unknown species	↑Se in the flower heads. ↑Se content in all parts of the plants.	(57)
*Brassica oleracea* L. (var. Capitata, cv. Pandion) and *Brassica oleracea* L. (var. Capitata, f rubra, cv. Erfurtskorano)	sodium selenate	20 mg of Se L^–1^ (Pandion), 0.5 mg of Se L^–1^ (f rubra)	field experiment (foliar application Pandion, soil fertilized twice, f rubra)	Pandion: in stems (μg of Se g^–1^ DW), 5.45. F rubra: in stems (μg of Se g^–1^ DW), 0.81	(ng of Se g^–1^ of sample): ↑SeMet Pandion in stems, 820; f rubra in stems, 200	↑Se content ↑SeMet	(58)
*Brassica oleracea* L. (var. Italica, cv. Monaco)	sodium selenate	young plants: weekly selenate applications of 0.8 μmol plant^–1^ via the root	young plants, 2 weeks after transplant (soil application, pot experiment, sand substrate)	in the adult plant heads (mg of Se kg^–1^ DW): upper stems, 5.5–58.0; terminal florets, 10–57.0	NA^c^		(137)
		adult plants: single foliar selenate application of 25.3 or 253 μmol plant^–1^	adult plants, 3-month old (field experiment, foliar application)				
*Brassica oleracea* L. (var. Capitata, f rubra, cv. Erfurtskorano)	sodium selenate	1st group: with a solution at a concentration of 2 μg of Se L^–1^ every second day for 2 months	field experiment (soil substrate)	in stems (ng of Se g^–1^ DW): 25–810. In leaves (ng of Se g^–1^ DW): 20–960	NA	≈anthocyanins, ≈chlorophyll	(60)
		2nd group: fertilized with 0.5 mg of Se L^–1^ twice in the same test period					

a FW: fresh weight.

b DW: dry weight.

c NA: not analyzed.

Interestingly, of the beneficial Se amino acids, SeMetSeCys was the only one identified in radish plants. This compound has recognized anticarcinogenic properties; thus its accumulation in radish roots is a valuable result. Plants sprayed with Se produced more SeMetSeCys compared to plants grown in hydroponics. The contents of Cys, polyphenols, and glutathione in Se-treated plants were higher than in the untreated plants. Concerning cabbage, both the total Se content and some nutritional traits of the edible parts increased after Se biofortification; in florets, Bañuelos et al.^56^ found higher percentages of Se organic compounds (such as SeMet and MeSeCys) than those of Se inorganic compounds. Also, Šindelářová et al.^57^ found the presence of Se organic compounds, such as SeMet and SeMetSeCys, in all the parts of the Se-biofortified plants and reported that Se accumulated mainly in the flower heads. Mechora et al.^58^ reported that the main soluble species in the Se-biofortified plants was SeMet, even if the major amount of Se was in insoluble forms (31–53%). Ramos et al.^59^ reported that half of the total Se accumulated in leaves was SeMetSeCys and SeMet, the total glucosinolate contents were not affected by the concentration of selenate application, and the total antioxidant capacity of plants was greatly stimulated by selenate. Mechora et al.^60^ reported that selenate addition had no effect on the amounts of anthocyanins or chlorophyll. Leafy crops are the most suitable for fortification studies; they require little time to reach maturity, they can be grown in pots, and they easily allow for the evaluation of the dose of the element that will be present in the edible part. Among all the leafy crops mentioned above, the most suitable for Se biofortification seem to belong to the Brassicaceae family. Since these are Se-hyperaccumulating plants, the main concern could be the risk of excessive doses of Se in the edible parts. However, as demonstrated by the total Se concentration values found by Mechora et al.^58,60^ and Šindelářová et al.^57^ on cabbage grown in fields and fertilized with Se by soil addition or foliar spray, it should not be difficult to develop an agronomic methodology to obtain leaves or plant heads with the right dose of Se. These edible parts contain, in addition to Se in inorganic forms, Se in organic forms (SeMet and SeMetSeCys), which are more easily available to the consumer.^57,58,60^

Se-biofortification studies were also carried out on plants whose edible parts were tuber, bulb, or root (potato, garlic, shallot, and carrot), and the obtained results are reported in Table 6.

**Table 6:** Bulb and Root Crops: Crop Species, Se Treatment (Se Source, Dose, and Application Mode), and Effects on Total (TseC) and Organic Se Content and Other Nutritional Traits

species	Se source	dose	type of treatment	TSeC	Se organic forms	other nutritional traits	references
*Solanum tuberosum* L. (cv. E potato-10)	sodium selenate	100 mg of Se L^–1^ and the final volume of the solution applied was 2 L plot^–1^	field experiment (foliar spraying)	in tubers (mg of Se kg^–1^ DW^a^): 0.055–1.05 (selenite), 1.04–1.50 (selenate)	↑SeMet (the main specie), ↑SeMeCys, ↑SeCys2	↑Se concentration	(34)
	sodium selenite						
*Solanum tuberosum* L. (cv. Agata)	sodium selenate	0.75–5.0 mg of Se kg^–1^	pot experiment (soil fortification)	in shoots (mg of Se kg^–1^ DW): 6.20 (selenite), 5.63 (selenate)	NA^b^	↑Se content	(63)
	sodium selenite			in tubers (mg of Se kg^–1^ DW): 5.0 (selenite), 10.0 (selenate)			
*Solanum tuberosum* L. (cv. Karin and Cv. Ditta)	sodium selenite	200–400 g of Se Ha^–1^	field experiment (foliar application)	in tubers (mg of Se kg^–1^ DW):1.562–2.027 (Karin), 0.693–1.129 (Ditta)	NA	↑content of total essential and nonessential amino acids	(138)
*Solanum tuberosum* L. (cv. Desiree)	sodium selenate	10 mg of Se L^–1^	field experiment (foliar application)	in tubers (ng of Se g^–1^ DW): 347 (drought exposed), 1101 (well-watered)	↑SeMet (68% of total Se)	↑selenate	(61)
*Solanum tuberosum* L. (cv. Satu)	sodium selenate	0.073–0.3 mg of Se kg^–1^ sand	in quartz sand (Se applied to the substrate)	in roots (μg of Se g^–1^ DW): 5–30	NA	≈starch concentration, ↑Se content	(62)
				in stolons (μg of Se g^–1^ DW): 4–40			
*Solanum tuberosum* L. (cv. Primura)	sodium selenate	50–150 g of Se Ha^–1^ in aqueous solution and in humic acid solution.	field experiment (soil substrate, foliar application)	in tubers (mg of Se kg^–1^ FW):0.01–0.15 (selenate), 0.01–0.11 (selenite) in aqueous solution.	NA	↑Se concentration	(30)
	sodium selenite			in humic acid solution: 0.01–0.35 (selenate)			
*Allium sativum* L.	sodium selenate	20.0–50.0 g of Se Ha^–1^	field experiment (foliar spray, FS; soil flood application, SFA)	in bulbs (mg kg^–1^ DW): 3.23 (highest average concentration)	NA	↑Se content, ↑total phenolics, ↑total flavonoids, ↑total antioxidant capacity	(64)
*Allium cepa* L. (aggregatum group, cv. Alba)	sodium selenate	63 mg of Se m^–2^, 50 mg L^–1^ SeCys solution	field experiment (foliar spray)	the inoculation of shallot plant roots with AMF increased the bulb Se content by 530%, and Se biofortification with (SeCys)_2_ and sodium selenate increased this value by 36% and 21%, respectively, compared to control	NA	↑ascorbic acid, ↑antioxidant activity	(65)
	selenocystine solution		Some plots were previously treated with an arbuscular mycorrhizal fungi (AMF)-based formulate				
*Daucus carota* (cv. Mokum F1)	sodium selenate	10 and 100 μg of Se mL^–1^	pot experiment (foliar spray)	in roots (μg g^–1^ DW): 0.5–2.2 (selenate), 0.4–1.5 (selenite)	↑SeMet, ↑γ-glutamil-SeMet-SeCys	↑Se content in roots and leaf	(66)
	sodium selenite						

a DW: dry weight.

b NA: not analyzed.

As far as the nutritional benefits are concerned, selenate was the most efficient source for Se biofortification of tubers;^34^ the accumulation of inorganic Se was higher in tubers treated with selenate (31.9% of the total Se content) than in those treated with selenite (1.5%).^34^ However, selenate was markedly inferior to selenite in terms of the organic transformation rate of Se.^34^ Selenate and SeMet were the main soluble Se species in potato tubers.^61^ In tubers, plant application of Se increased the relative content of total essential and nonessential amino acids compared to the controls (phenylalanine was enhanced particularly).^62^ When applied in small doses, Se provided beneficial effects on the tuber production, activated enzymes of the antioxidant system,^63^ and delayed aging of the stolons and roots, contributing to an increased shelf life of potatoes.^61^ At harvest, the starch concentration in tubers did not change.^61^ In garlic, foliar spray was more effective than soil application. A significant increase in total phenolics, total flavonoids, and total antioxidant capacity was observed in bulbs.^64^ Concerning shallots, it was reported that Se biofortification combined with pretreatment of an arbuscular mycorrhizal fungi (AMF)-based formulate increased the bulb Se content by 530%, while Se biofortification with selenocystine (SeCys_2_) and selenate increased this value by 36% and 21%, respectively, compared to the control. The values of bulb quality indicators, macro- and microelements, ascorbic acid, and antioxidant activity increased upon AMF inoculation;^65^ both selenite and selenate positively affected most of the quality attributes and macroelements as well as the contents of Se and ascorbic acid. For carrots, inorganic Se, SeMet, and γ-glutamil-SeMet-SeCys were the predominant Se forms in roots.^66^

In Italy, potatoes, onions, and carrots containing low concentrations of Se (suitable for human diet) are already in trade and are produced by the Italian Potatoes of Quality Consortium, with headquarters in Bologna.^30^ Since tubers, bulbs, and roots are poor but nutritious foods, improving their nutritional characteristics even by increasing their content of Se in organic forms appears relevant for the wellness of populations of the poorest areas of the world. .

As far as fruit vegetables are concerned, the plant most commonly used in Se biofortification studies was tomato, whose results are reported in Table 7. Biofortification with Se seemed to cause a delay in the onset of the fruit ripening.^54,67,68^ This effect may be positive because it could affect the postharvest shelf life of tomatoes; Zhu et al.^67^ reported that this could be due to an inhibition of reactive oxygen species (ROS) generation by stimulation of antioxidant defense systems, together with a downregulation of ethylene biosynthesis genes. Similarly, Puccinelli et al.^54^ noticed a lower respiration rate and ethylene production, associated with a delayed lycopene and β-carotene synthesis and chlorophyll degradation. The nutritional benefits that tomato fruits acquired with Se biofortification were the presence of SeMet and MetSeCys as the major forms of Se compounds in the fruits,^69^ an increase of the antioxidant activity,^70,71^ a slightly higher level of vitamin A,^45^ and an increase in fruit firmness and fruit total solids.^70^ Se biofortification of tomatoes may be interesting for fortified food producers. Also, in this case, it is essential to develop an agronomic method that allows fruits to be obtained with a dose of Se suitable for the human diet. Particularly interesting, from this point of view, is the fortification technique developed by Businelli et al.,^45^ which is as follows: (i) enrich an appropriate amount of peat in Se, (ii) sow the seeds of the selected crop species in Se-enriched peat until seedlings have the appropriate size for transplanting, (iii) transfer these Se-enriched transplants in the field. Moreover, using this technique, the environmental spread of Se is minimized, as this element is not in any way distributed in the field, but it is only used during the pre-transplanting stage and is immediately absorbed by the seedlings. Another on-field fortification technique, suitable for obtaining a Se-fortified tomato without excessive Se concentrations, is that proposed by Andrejiová et al.^72^ The Se fortification of tomatoes has potential for obtaining a table fruit with a longer shelf life and with high levels of Se-organic forms and antioxidant compounds. Another possible use could be the production of sauce; in this case, Se-fortified tomatoes could be mixed with untreated tomatoes in order to avoid excessive Se concentrations in the final product.

**Table 7:** Tomato: Plant Genotype, Se Treatment (Se Source, Dose, and Application Mode), and Effects on Total (TSeC) and Organic Se Content and Other Nutritional Traits

species	Se source	dose	type of treatment	TSeC	Se organic forms	other nutritional traits	references
*Solanum lycopersicum* L. (cv. Red Bunch)	sodium selenate	1–1.5 mg of Se L^–1^	in hydroponics	in fruits (mg kg^–1^ DW^a^): 0.94–2.76 (1 mg L^–1^ treatment), 2.08–3.54 (1.5 mg L^–1^ treatment)	NA^b^	↑delayed fruit ripening, ↑shelf life, ↑delayed lycopene and *β*-carotene synthesis, ↑chlorophyll degradation	(54)
*Lycopersicon esculentum* Mill. (var. Durpeel and var. Uno Rosso F1)	sodium selenate	150 g of Se Ha^–1^ (at the flowering stage)	field experiment (foliar application)	in fruits (mg kg^–1^ DW): 0.378 (Durpeel)–0.990 (Uno Rosso F1)	NA	↑Se content in fruits, ≈total carotenoids, ≈vitamin C, ↑total polyphenols	(72)
*Solanum lycopersicon* L. (cv. Provence)	sodium selenate	1 mg of Se L^–1^ (at the onset of flowering)	Green house experiment (foliar application)	Not reported	NA	↑delayed fruit ripening	(67)
*Lycopersicon esculentum* Mill. (var. Toro)	sodium selenite	5 and 10 mg of Se L^–1^ (nutrient solution)	pot experiment (peat moss and perlite substrate, Se with the nutrient solution)	in fruits (μg g^–1^ DW): 24.0–33.0	NA	↑fruit firmness; ↑total solids; ≈N, P, K, Ca, and Mg; ↑antioxidant enzyme activities	(70)
*Solanum lycopersicum* L. (cv. Karst)	sodium selenate	1–50 mg of Se kg^–1^ of peat.	in pots (peat substrate, greenhouse experiment)	in edible organs, open field experiments (μg of Se kg^–1^ DW):15.4–19.7 (in 2012), 14.9–20.2 (in 2013)	NA	↑Se content, ↑vitamin A	(45)
			some plants grown and Se-fortified in pots were transplanted in open field				
*Solanum lycopersicum* L.	sodium selenate	2.0–10.0 mg of Se L^–1^ solution.	in pots (greenhouse experiment): soil application + foliar spray (SF) seed soaking (SS)	Not reported	NA	↑antioxidant activities	(71)
*Solanum lycopersicum* L. (cv. PKM. 1)	sodium selenate	2.0–10.0 mg of Se L^–1^ solution.	in pots (greenhouse experiment): soil application + foliar spray (SF) seed soaking (SS)	in fruits (μg of Se g^–1^ DW): 26.52–52.24	in fruits: ↑SeMet, ↑MeSeCys	↑total phenolic, ↑total protein, ↑nitrate, ↑total antioxidant activity, ↓chlorophyll, ↑Se concentrations	(69)
*Solanum lycopersicum* L. (cv. Red Bunch)	sodium selenate	1.0 mg of Se L^–1^ (in the nutrient solution 2 weeks after transplanting)	in greenhouse (plants hydroponically grown and then transplanted into rock wool slabs)	in fruits (μg of Se g^–1^ DW): 10.28–11.46	NA	↑Se content, ↓β-carotene content, ↓ethylene, ↑delay in the onset of fruit ripening	(68)

a DW: dry weight.

b NA: not analyzed.

### Microscale Vegetables

Recent studies on Se biofortification were focused on “microscale vegetables”, i.e., plants in early growth stages, since they are able to absorb relevant amounts of Se^73^ and are naturally rich in phytochemicals.^74−76^ Microscale vegetables differ from each other according to their corresponding growing cycle lengths, plant heights, edible portions, and other secondary traits.^74,76^ This section will review only literature on sprouts (i.e., 3–5 day-old seedlings), grasses (7–12 day-old seedlings from *Graminaceae* species), and microgreens (5–10 day-old seedlings from all plant species except for *Graminaceae* species). These require a short time interval to be produced (1–3 weeks) and few inputs (i.e., no soil, only water, and no or low light).^74,76^Tables 8–10 report the studies of the last ten years that concern the most exploited technique for Se biofortification in sprouts, grasses, and microgreens: Se is supplied by (i) the germination substrate (Table 8), (ii) the soaking procedure (Table 9), and (iii) the chemical priming (Table 10). All the tables report the effect of these methods on total and organic Se content and, where studied, on phytochemicals.

**Table 8:** Microscale Vegetables: Plant Species, Growth Stage, and Se Treatment (i.e., Se Source, Se Doses, and Time of Exposition) with Se Applied to the Germination Substrate

species	growth stage (DAS)^a^	Se source	dose	TSeC	organic Se	other nutritional traits	reference
Graminaceae							
*Oryza sativa* (rice)	10	sodium selenate	5, 10, 15, and 20 mg of Se L^–1^	300–500 mg kg^–1^ DM^b^	SeMet, SeCys_2_, SeMetCys	↑PAs (free and conjugated), ↓carotenoids	(77)
	10	sodium selenite	5, 10, 15, 20 and mg of Se L^–1^	300–500 mg kg^–1^ DM	SeMet, SeCys_2_, SeMetCys	↑PAs (free and conjugated), ↓carotenoids	(77)
	8	sodium selenite	10, 20, 30, and 40 mg of Se L^–1^	10–25 mg kg^–1^ DM	NA^c^	≈polyphenols	(139)
	1–4	sodium selenite	10, 20, 30, and 60 μM	∼2 and 8 μg g^–1^ DM	NA	NA	(78)
*Secale cereale* (rye)	7	Se oxide	10 mg of Se L^–1^	53 μg g^–1^ DM	NA	↓antioxidant activity, ≈GLS^d^	(80)
Leguminosae							
*Lupinu sangustifolius* (lupin)	5	sodium selenite	2, 4, 6, and 8 mg L^–1^	∼1–5 μg g^–1^ DM	NA	↑antioxidant acitivity	(140)
	5	sodium selenate	2, 4, 6, and 8 mg L^–1^	∼2–14 μg g^–1^ DM	NA	↑antioxidant acitivity	
*Medicago sativa* (alfalfa)	21	sodium selenite sodium selenate	1, 2.5, and 4 mg of Se L^–1^	132–284 mg kg^–1^ DM	SeCys_2_, SeMet	NA	(83)
*Lens culinaria* (lentil)	21	sodium selenite sodium selenate	1, 2.5, and 4 mg of Se L^–1^	98–111 mg kg^–1^ DM	SeCys_2_, SeMet	NA	(83)
*Glycine max* (soy)	21	sodium selenite sodium selenate	1, 2.5, and 4 mg of Se L^–1^	158–188 mg kg^–1^ DM	SeCys_2_, SeMet	NA	(83)
Brassicaceae							
*Brassica oleracea* (var. italica) (broccoli)	15	sodium selenite	20 μM	801–1789 μg g^–1^	SeMetCys, SeMet	↑antioxidant acitivity, ↑GLS in some varieties	(59)
	7	sodium selenite	10, 25, 50, 75, and 100 μM	20–185 μg g^–1^ DM	SeMetCys	↓glucoraphanin	(79)
	7	sodium selenate	10, 25, 50, 75, and 100 μM	32–263 μg g^–1^ DM	SeMetCys	≈GLS	(79)
	8	sodium selenate	50 μM	132 μg g^–1^ DM	NA	↑antioxidant activity and phenolics	(141)
	5	sodium selenite	100 μM	70 μg g^–1^ DM	NA	↓≈polyphenols, ↑anthocyanins, ↑flavonoids, ≈GLS (↑sulphoraphane)	(81)
	5	sodium selenate	100 μM	85 μg g^–1^ DM	NA	↓≈polyphenols, ↑anthocyanins, ↓≈flavonoids, ≈GLS (sulphoraphane variable among cultivars)	(81)
	7	sodium selenate	50 μM	160 μg g^–1^ DM	SeMeCys	≈GLS	(79)
	7	sodium selenate	30, 60, 90, 120, and 150 mg of Se L^–1^	467 mg kg^–1^	SeMetSeMeCys	NA	(82)
	7	Se oxide	10 mg of Se L^–1^	400 μg g^–1^ DM	NA	↓antioxidant activity, ≈GLS content	(80)
*B. oleracea* (var. botrytis) (cauliflower)	7	sodium selenate	50 μM	150–230 μg g^–1^ DM	SeMeCys	↑≈total and single GLS depending on varieties	(79)
*B. olearacea* (var. acephala) (kale)	7	sodium selenate	50 μM	140–320 μg g^–1^ DM	SeMeCys	≈GLS	(79)
*B. oleracea* (var. gemmifera) (Brussels sprouts)	7	sodium selenate	50 μM	80 μg g^–1^ DM	SeMeCys	≈GLS	(79)
*B. oleracea* (var. capitata) (cabbage)	7	sodium selenate	50 μM	180 μg g^–1^ DM	SeMeCys	≈GLS	(79)
*B. rapa* (ssp. pekinensis) (Chinese cabbage)	7	sodium selenate	50 μM	160–310 μg g^–1^ DM	SeMeCys	≈GLS	(79)
*B. chinensis* (var. pekinensis) (packchoi)	7	sodium selenate	30, 60, 90, 120, and 150 mg of Se L^–1^	312 mg kg^–1^	SeMetSeMeCys	NA	(82)
*B. albogabra* (kale)	7	sodium selenate	30, 60, 90, 120, and 150 mg of Se L^–1^	156 mg kg^–1^	SeMetSeMeCys	NA	(82)
*B. oleracea* (var. *capitata* f. *alba*) (white cabbage)	7	Se oxide	10 mg of Se L^–1^	382 μg g^–1^ DM	NA	↑antioxidant activity ≈GLS content	(80)
*Sinapis alba* (mustard)	7	selenium oxide	10 mg of Se L^–1^	138 μg g^–1^ DM	NA	↑antioxidant activity, ≈GLS	(80)
*Lepidium sativum* (garden cress)	5	sodium selenite	4 and 8 mg of Se L^–1^	21–36 μg g^–1^ DM	NA	↑antioxidant acitivity, ↑GLS	(140)
	5	sodium selenate	4 and 8 mg of Se L^–1^	27–39 μg g^–1^ DM	NA	↑antioxidant acitivity, ↑GLS	(140)

a DAS: days after sowing.

b DM: dry matter.

c NA: not analyzed.

d GLS: glucosinolate content.

**Table 9:** Microscale Vegetables: Plant Species, Growth Stage, and Se Treatment (i.e., Se Source, Se Doses, and Time of Exposition) with Se Applied by Soaking

species	growth stage (DAS)^a^	Se source	dose	time	TSeC	organic Se	other nutritional traits	reference
Leguminosae								
*Cicer arietinum* (chickpea)	1–4	sodium selenite	1 and 2 mg in 85 mL of water	6 h	4–7 μg g^–1^ DM^b^	NA^c^	↑antioxidant activity, ↑total isoflavones, ↑some single isoflavone	(142)
*Medicago sativa* (alfalfa)	∼5, 7	sodium selenite	1 and 10 mg of Se L^–1^	6–10 h	13–109 mg kg^–1^ DM	SeMet, SeMetSeCys	NA	(80)
*Vigna radiata* (mung bean)	3, 5	sodium selenate	127, 1270, and 12700 μM	10 h	up to 200 μg g^–1^ DM	NA	NA	(143)
	3	sodium selenite	0–12 mg of Se L^–1^	24 h	571–7275 μg kg^–1^	SeMetSeCys	NA	(144)
Brassicaceae								
*B. oleracea* (var. *italica*) (broccoli)	11	sodium selenate	10, 50, and 90 μM	4 h	NA	NA	≈polyphenols, ↓quercetin and sinapic acid, ↑morine and genisteine	(145)
	∼5, 7	sodium selenite	1 and 10 mg of Se L^–1^	6–10 h	∼22–133 mgkg^–1^ DM	SeMet, SeMetSeCys	NA	(80)
	3, 5	sodium selenate	127, 625, and 1270 μM	10 h	∼250 μg g^–1^ DM	NA	NA	(143)
*B. oleracea* (var. *capitata*) (red cabbage)	∼5, 7	sodium selenite	1 and 10 mg of Se L^–1^	6–10 h	13–82 mg kg^–1^ DM	SeMet, SeMetSeCys	NA	(80)
*Raphanus sativus* (var. *sativus*) (radish)	∼5, 7	sodium selenite	1 and 10 mg of Se L^–1^	6–10	10–103 mg kg^–1^ DM	SeMet, SeMetSeCys	NA	(80)
*R. sativus* (var. *longipinnatus*) (daikon sprouts)	∼5, 7	sodium selenite	1 and 10 mg of Se L^–1^	6–10 h	13–97 mg kg^–1^ DM	SeMet, SeMetSeCys	NA	(80)
*Sinapis alba* (white mustard)	∼5, 7	sodium selenite	1 and 10 mg of Se L^–1^	6–10 h	12–78 mg kg^–1^ DM	SeMet, SeMetSeCys	NA	(80)
other								
*Allium cepa* (onion)	5, 7	sodium selenate	127, 625, and 1270 μM	10 h	up to 600 μg g^–1^ DM	NA	NA	(143)
*Amaranthus cruentus*, *A. caudatus*, *A. paniculatus*, and *A. tricolor* (amaranth)	6	sodium selenite	10, 15, and 30 mg L^–1^	3 h	35–80 mg kg^–1^ DM	NA	≈antioxidant activity (FRAP), ≈↓DPPH	(146)
*Fago pyrum esculentum* (buckwhaet)	11	sodium selenite	10 mg of Se L^–1^	4 h	2 μg g^–1^ DM	SeMet	NA	(147)
	11	sodium selenate	10 mg of Se L^–1^	4 h	7 μg g^–1^ DM	SeMet	NA	(147)
	11	SeMet	10 mg of Se L^–1^	4 h	3 μg g^–1^ DM	SeMet	NA	(147)

a DAS: days after sowing.

b DM: dry matter.

c NA: not analyzed.

All the procedures used for Se biofortification generally cause an increase of Se content, but results varied with the species; the growth stage; and the Se source, dose, timing of application.

The growth stage should be chosen accurately since it is related to the edible portion of the plant. In the case of sprouts, the whole seedling (shoots and roots) is edible, while in the case of microgreens and grass, only the shoot is used in human nutrition (i.e., for salads, soups, or juices).^75,76^

The organ to be consumed may also depend on the form of Se used for biofortification. In fact, by using sodium selenite (Na_2_SeO_3_), the Se might be highly accumulated in the roots (i.e., mainly as selenite), while by using sodium selenate (Na_2_SeO_4_), the Se will be accumulated mainly in the shoots as selenate and organic Se.^29,77^

The Se source used for biofortification is strongly related to the chemical form of Se consumed by nutrition. On the other hand, the chemical product containing Se is often chosen according to cost-effective parameters. Within the existing compounds suitable for Se biofortification, inorganic ones (e.g., sodium selenite and sodium selenate) are known to be cheap and efficient, whereas organic ones (i.e., selenoamino acids) are expensive but more relevant for human nutrition.^77^ Since plants are able to produce selenoproteins starting from inorganic Se compounds, inorganic forms are the most preferred for Se biofortification,^77^ as demonstrated by scientific literature reported in Tables 8–10.

**Table 10:** Microscale Vegetables: Plant Species, Growth Stage, and Se Treatment (i.e., Se Source, Se Doses, and Time of Exposition) with Se Applied by Priming

species	growth stage (DAS)^a^	Se source	dose	time	TSeC	organic Se	phytochemicals	reference
*Oryza sativa* (rice)	5, 10	sodium selenite	0.8 and 1 mg of Se L^–1^	24 h	NA^b^	NA	↓polyphenols	(148)
	18	sodium selenite	15, 30, 45, 60, 75, 90, and 105 μmol of Se L^–1^	24 h	NA	NA	≈polyphenols (slight increase at the highest Se dose)	(149)
	7	not specified	60 μM Se	24 h	NA	NA	NA	(150)
	18	not specified	60 μM Se	24 h	NA	NA	↑antioxidant activity	(151)
*Triticum aestivum* (wheat)	18	sodium selenate	0, 25, 50, 75, and 100 μM Se	30 min	NA	NA	NA	(152)

a DAS.: days after sowing.

b NA: not analyzed.

As far as the Se dose is concerned, studies are needed to individuate the optimal dose, i.e., the dose that increases Se accumulation and phytochemical concentration without compromising seedling growth in order to maximize the yield of total and organic Se and of phytochemicals. It should be noted that very high Se doses are not worthwhile since they depress plant growth and may cause very high Se concentrations, which may limit the consumption of microscale vegetables in order to not exceed the recommended daily Se intake.

Finally, the method and time of exposure for Se biofortification treatment significantly affect the final results in terms of Se and phytochemical contents. Concerning Se application via the germination solution, the common procedure consists of sowing seeds on the substrate containing different solutions of Se until the day of harvest (Table 8). Since the germination period may vary between 5 and 15 days, the solution in the substrate has to be restored often, especially when the trays for sprouting are open. Some authors added a specific volume of the corresponding Se solution to restore the solution content,^78,79^ and others sprinkled or sprayed the Se solution at specific times.^80,81^ When possible, due to the long duration of the germination period (i.e., 1521 days), some authors changed the nutrient solution containing Se.^59^ The choice is also affected by the presence^78,82^ or absence^77,83^ of the substrate (i.e., paper, sand, etc.). Different procedures imply differences in the evolution of Se concentration in the germination substrate, and as a consequence, the results in the literature are often not comparable.

Considering the soaking (Table 9) and priming with Se (Table 10), the main variations are due to the time of exposition to the treatment. In the case of soaking, the time of treatment may vary from 4 to 24 h depending on the size of seeds, and Se content generally increases with increasing time of exposition. Studies on priming with Se did not report results concerning the content of total Se and Se proteins, probably because these studies were more focused on plant growth parameters and stress resistance than on nutritional traits.

In addition to the aforementioned techniques, the recent work of Puccinelli et al. is noteworthy,^84^ in which they reported the possibility of producing Se-enriched sprouts from seeds harvested by a mother crop fertilized with Se. This might represent an innovative method to produce Se-enriched microgreens.

### Fruit Tree Crops

Despite the considerable knowledge of Se effects and accumulation on herbaceous species, little is known about trees species. In particular, the present section will focus just on Se effects on fruits and their derivates, as little evidence has been reported on Se accumulation especially in the edible fruits and their derivates (juice, wine, and oil) (Table 11). The content of Se in tree plants can be increased in different ways, including soil and foliar fertilization. From the bibliography examined, it emerges that the most used modality for Se biofortification in tree plants is the foliar spray. In general, foliar spraying was preferable in comparison to soil application, since it involves a more efficient uptake of Se, an absence of residual effects, and a minimum consumption of Se salts, resulting in the most environmentally safe and economically acceptable method.^31,85^ A little-explored treatment modality is that of fruit treatment. Pezzarossa et al.^86^ investigated the effects of foliar and fruit spraying of sodium selenate on Se accumulation, fruit growth, and senescence in peach and pear fruit crops. Both treatments increased the fruit Se concentration, but fruit treatment was more effective than leaf treatment in increasing Se content in fruits. The daily consumption of pears and peach treated with 1 mg of Se L^–1^ does not induce toxicity but can even provide a rational Se supplementation for human nutrition. Se accumulated in the pear juice was almost all inorganic, so the application of selenite is considered more suitable than selenate from the viewpoint of food safety.^87^ In apples and pomegranates, Se supplementation via foliar spray enhanced fruit quality.^88,89^ In particular, in apples, in addition to the increase of Se content, an increase in the flesh firmness, titrable acidity, soluble solid content, and activities of antioxidant enzymes were observed,^90^ while in pomegranates, Se fertilization led to an important increase of the content of phenolic compounds, antioxidants, and anthocyanins.^89^

**Table 11:** Fruit Tree: Crop Species and Genotype, Se Treatment (Se Source, Dose, and Application Mode), and Effects on Total (TSeC) and Organic Se Content and Other Nutritional Traits

sample	Se source	dose	type of treatment	Se content	TSeC	Se organic forms	other nutritional traits	reference
*Olea europaea* L. (cv. Leccino)	sodium selenate	100 mg of Se L^–1^	leaves spray	↑oil	up to 120 μg of Se kg^–1^	NA^a^	↑phenols content in the oil, ↑PAL activity	(93)
*Olea europaea* L. (cv. Leccino)	sodium selenate	100 mg of Se L^–1^	leaves spray	↑fruits	6.1 μg of Se g^–1^	NA	↑B, Mg, K, Cr, Mn, Fe, and Cu in edible parts	(91)
*Olea europaea* L. (cv. Maurino)	sodium selenate	50 and 150 mg of Se L^–1^	leaves spray	↑oil	430.8–956.6 μg of Se kg^–1^	NA	↑pigment, ↑phenol content, ≈fruit characteristics, ≈sensory quality of the oil	(31)
*Olea europaea* L. (cv. Leccino)	sodium selenate	100 mg of Se L^–1^	leaves spray	↑Se content in extra virgin olive oil	171–529 μg of Se kg^–1^	SeMet	↑phenol, carotenoid, and chlorophyll	(153)
*Vitis vinifera* L. (cv. Hutai no. 08)	Amino acid-chelated	Se ≥ 0.12 g L^–1^	leaves spray	↑fruits	22.90 μg of Se kg^–1^	NA	↑acid invertase activity, ↑total soluble sugar and Se content in berries	(96)
*Vitis vinifera* L. (cv. Sangiovese)	sodium selenate	100 mg of Se L^–1^	leaves spray	↑fruits and wine	0.800 ± 0.08 mg of Se kg^–1^ (DM^b^) in the grapes; 0.620 ± 0.09 mg of Se L^–1^ in wine	52.5% of the total Se	↑Se content	(97)
*Vitis vinifera* L. (cvs. Crimson Seedless, RedBarbara, Summer Black, and Hutai no.8)	amino acid-chelated Se	organic Se content ≥60 g L^–1^ (diluited 500 times)	leaves spray	↑fruits	19.46–34.96 μg of Se kg-1 (FW^c^)	NA	↑soluble sugar; ↑Vc; ↑soluble protein; ↑soluble solid; ≈polyphenol; ↑K and Ca; ↓Pb, Cr, Cd, As, Ni	(95)
*Malus domestica* Borkh. (cv. Starking Delicious)	sodium selenate	0, 0.5, 1, and 1.5 mg of Se L^–1^	leaves spray	↑fruits	0.1 μg of Se kg^–1^	NA	↑flesh firmness, titrable acidity, and soluble solid content; ↑activities of antioxidant enzymes	(88)
*Prunus Persica* L. Batsch (cv. Flavorcrest and cv. Suncrest) and *Pyrus communis* L. (cv. Conference)	sodium selenate	0.1 and 1.0 mg of Se L^–1^	leaves (LT) and fruits spray (FT)	↑fruits	33–199 μg of Se kg^–1^	NA	↑fruits flesh firmness, ↑soluble solid content	(86)
*Pyrus communis* L. (cv. Liuyuexueli)	sodium selenite and sodium selenate	20, 40, 50, 100, 200 mg of Se L^–1^	leaves spray	↑fruits	selenate treated > selenite treated	70–80% Se transformed in organic form	<40 mg L^–1^ optimal Se concentration and Se(IV) more suitable (food safety)	(87)
*Punica granatum* L. (cv. Malase Saveh)	sodium selenate and Se nanoparticles	1 and 2 μM	leaves spray	↑leaves	1.5–2.5 μg Se g^–1^	NA	↑peel thickness, ↑phenolic compounds, ↑antioxidants, ↑anthocyanins	(89)

a NA: not analyzed.

b DM: dry matter.

c FW: fresh weight.

Regarding the effects of Se supplementation (100 mg L^–1^ via foliar spray) in table olives, D’Amato et al.^91^ reported that, at harvesting time, the concentration in the edible part of the drupes delivered 6.1 μg g^–1^, corresponding to 29 μg of Se per 5 olives (39 and 49% of the recommended dietary allowance (RDA) for adult men and women, respectively), and such enrichment also changed the nutritional quality of the drupes, with significant increases in the concentrations of B, Na, Mg, K, Cr, Mn, Fe, and Cu compared to the untreated control group. Therefore, in addition to Se, the consumption of 10 g of Se-enriched olives (five olives) per day per person would provide a quantity of Cu, K, Fe, Mg, Mn, and Zn equal to 3, 9, 1, 1, 1, and 0.5% of the RDA, respectively.^92^

Se fertilization via foliar spray (50, 100, and 150 mg L^–1^) is also effective for the enrichment of extra virgin olive oil (EVOO) in Se content (up to 120 μg kg^–1^).^31,93^ Moreover, Se fertilization increased SeMet, carotenoid, chlorophyll, and phenol content in EVOO.^93,94^ In particular, the phenolic profiles showed that oleacein, ligustroside aglycone, and oleocanthal were the most affected compounds and were increased by 57, 50, and 32%, respectively. All these compounds, especially oleacein, have been shown to exert a relevant antioxidant activity, contributing to both the shelf life of EVOOs and positive effects on human health.^93^ It is important to underline that foliar spray with Se may be particularly useful with EVOOs characterized by a poor phenolic profile, which cannot meet the European Food Safety Authority (EFSA) statement about the admissibility of the health claim for EVOOs. Indeed, a well-planned Se fertilization before flowering may help these EVOOs reach the minimum content of hydroxytyrosol and its derivatives (e.g., the oleuropein complex and tyrosol).

In vitis grapes, the acid invertase activity, total soluble sugar, and Se content produced by plants treated with Se amino-acid-chelated fertilizer were higher than in the untreated control. In addition, Se fertilizer improved the nutritional characteristics, including soluble sugar, soluble protein, soluble solid, and reduced organic acid contents, while it had no effect on the polyphenol antioxidants of Eurasian species. Moreover, Se fertilization can be used not only to increase the Se content and nutrition quality of grapes but also to reduce the accumulation of heavy metals Pb, Cr, Cd, As, and Ni.^95,96^

Immediately after the malolactic fermentation of Se-enriched (100 mg L^–1^ via foliar spray) grape berries, the wine obtained from treated trees had a Se content of 0.620 ± 0.09 mg of Se L^–1^.^97^ In particular, the percentage of inorganic Se was 26% of the total Se in the untreated wine, while in Se-enriched wine, this percentage increased to 47.5% of the total Se. Selenite was the inorganic chemical form most present in enriched wine, probably due to the foliar application with selenate. Given a daily wine consumption of 50 mL, the contribution to the daily Se RDA is remarkable, since it is 91 and >100% for adult men and women, respectively, as considered by FAO/IAEA/WHO consultation, and 44 and 62% for adults, as considered by USDA. In addition, the amount of alcohol contained in a recommended volume of enriched Sangiovese wine is less than the quantity referred to the moderate wine consumption (15.5–31 g of alcohol day^–1^).

In general, foliar treatment with Se resulted in the effective enhancement of Se content in fruits (olives, grapes, pears, peachs, pomegranates, and apples) and their derivates (oil, wine, and juice) and their nutritional quality. However, the accurate planning of Se fertilization (time and dose) is necessary in order to avoid damage to the photosynthetic apparatus, inhibiting photosynthesis and the primary metabolism, and to maximize the protection from environmental stresses and the products quality.

## Selenium Supplementation in Livestock: Effects on Meat Quality

Se is an essential trace element in animal nutrition and exerts multiple actions related to performance, fertility, health, and product quality.^98^ Different forms of Se supplements are available for animal feed, and in particular, two major Se sources are used: inorganic (mainly selenite or selenate) and organic, mainly in the form of SeMet (mainly as Se yeast or SeMet preparations). Many factors can affect the activity and efficacy of Se supplementation, such as the chemical form, animal’s health, and environmental conditions. Both organic and inorganic forms are metabolized by animals, mainly as SeCys, which is the form in which Se is also consumed by humans (through animal-origin products).^99^ The body of literature has reported that dietary Se supplementation increases Se concentration in the meat of rabbits,^100^ lambs,^101^ calves,^102^ and chickens.^103,104^

Se is classified as an antioxidant microelement because it is a part of the active center of the enzyme glutathione peroxidase (GPx) as well as a cofactor for thioredoxin reductase^105^ in blood, liver, and edible tissues,^106^ which might be connected with enhancing the immune response in mammals. There were several documented reports that the addition of organic Se in animal feed resulted in enhanced GPx activity and oxidative stability of meat.^107^ Lipid oxidation is the main cause of deteriorating meat quality in terms of color, flavor, texture, and nutritional value.^108^ Joksimovic-Todorovic et al.^109^ reported that Se has an effect of preserving the texture and sensory characteristics of meat among domestic animals. Also, this type of supplementation induced a decrease in the fat and cholesterol contents in the meat (i.e., beef).^110,111^

Furthermore, Se may play a role in the alteration of lipid metabolism; a decrease of the content of cholesterol in meat when adding Se would be a beneficial effect of its supplementation. Nevertheless, the results concerning lipid decrease^111^ were not consistent with those reported in other studies in cattle,^112,113^ rabbit,^100,114^ or pigs,^115,116^ for which no difference was observed in lipid amount when adding Se. The Se source was reported to have no direct effect on the meat fatty acid profile; however, improving the oxidative stability of meat indirectly affected the lipid composition, thereby preserving the meat quality (Table 12).^101,114,117^ Such a discrepancy is mainly due to the form in which Se was administered; the organic Se is known to be linked to a higher Se content in the meat compared to the inorganic Se.^118^ However, SeMet, being incorporated into general proteins (methionine codon), results in greater availability than SeCys, demonstrating that it is easier to enrich meat with Se by providing animals with additional SeMet in their feed.^119^

**Table 12:** Livestock (Species, Breed, and Muscle), Se Treatment (Se Dose and Source), and Main Effects of Se Supplementation in Animal Feeding

species	breed	muscle	dose	Se source	main effects	reference
Beef						
	Limousin × Holstein–Friesian	longissimus dorsi and psoas major	0.30 or 0.50 mg of Se kg^–1^	Se-enriched yeast, sodium selenite	↑Se and GPx activity in meat, little or no effect in meat oxidative stability	(154)
	Charolais	longissimus thoracis	0.3 mg of Se kg^–1^	Se-enriched yeast, sodium selenite	↑Se concentrations for the Se yeast, ↑color lightness, ↓shear force	(113)
Pig						
	[Landrace × Yorkshire] × Duroc	loin	0.3 mg of Se kg^–1^	Se-enriched yeast, Se-proteinate	↓Se concentrations in loin for the Se yeast	(155)
	Duroc × Landrace × Yorkshire	longissimus dorsi	0.3 mg kg^–1^	Se-enriched yeast	↓drip loss; ↓lightness; =redness, TBARS, and thiols	(156)
Poultry						
	broiler	breast and leg	0.3 mg of Se kg^–1^	sodium selenite	↑color degree, ↓drip losses, ↑serum GPx	(157)
	ArborAcres	pectoralis major	0.3, 0.5, 1.0, or 2.0 mg of Se kg^–1^	nano-Se	↓TBARS, ↑muscle glutathione peroxidase	(158)
	Ross 308	breast	0.15 mg of Se kg^–1^	SeMet	↑total antioxidant capacity, ↓malondialdehyde concentration	(159)
	high line turkeys	pectoralis major and peroneus longus	0.08 or 0.23 mg of Se kg^–1^	seleno yeast, sodium selenite	↑muscle tissue GPx activities	(160)
Rabbit						
	Californian	hindleg	0.3 mg of Se kg^–1^	SeMet	↑vitamin E and Se; ↓index of lipid oxidation, TBARS	(161)
	New Zealand white	longissimus dorsi	10% of Se-fortified olive leaves (2.10 mg kg^–1^)	sodium selenate solution	↑oleic acid, ↓desaturase index, ↓TBARS	(117)
	New Zealand white	longissimus dorsi	10% of Se-fortified olive leaves (2.10 mg kg^–1^)	sodium selenate solution	↓TBARS, ↑GPx and α-tocopherol, ↑SeMet and SeCys_2_ in meat	(114)
	hyplus	loin and hindleg	0.12 mg of Se kg^–1^	Se yeast (Sel-Plex, Alltech)	↑Se content of meat	(100)
Lamb						
	Italian apennine lambs	longissimus dorsi	0.30 mg of Se kg^–1^	sodium selenite	↑Se content of meat	(162)
	lambs	longissimus dorsi	0.30 mg of Se kg^–1^	sodium selenite + Vit E	↓TBARS	(163)
	north country mule × Suffolk	longissimus dorsi	0, 0.11, 0.21, or 0.31 mg of Se kg^–1^	selenized enriched yeast, sodium selenite	no significant effects of treatment on meat quality assessments	(164)

## Perspectives for Future Research

To date, scientific research has aimed to identify the Se effects on the agronomic and physiological parameters of biofortified plants, so most of the literature reviewed here considered very high Se doses, which normally depress plant growth. This approach, however, is often incompatible with the aim of obtaining a Se-enriched food suitable for human and animal diet. Therefore, when the production of Se-enriched foods that provide nutritional benefits is the main goal of the research, it is necessary to carefully evaluate the applied Se-biofortification strategies and cost-effective parameters. In this regard, the challenge for future research on plant-food biofortification will be to fine-tune the fortification techniques in terms of the Se source and dose as well as the timing and modality of application, tailored for each plant species, growth stage, and cultivation condition. An abundance of the literature reviewed here considered Se hyperaccumulator plants and very high Se doses, which normally depress plant growth. Future research should focus on biofortification at lower Se doses, since this is expected to increase Se yield (i.e., the product between plant biomass and its Se concentration), and with organic rather than inorganic Se forms, while avoiding overabundant accumulation in plant foods, thus limiting the risk of exceeding the recommended dietary intake in humans. Finally, future research on the Se biofortification of plants will have to consider species that are scarcely exploited for food items but may be of interest in food supplementation and nutraceutics. An example is given by the Se enrichment of *Pueraria lobata*, whose roots were found to be high in Se-containing proteins and polysaccharides potentially useful as anticarcinogenic molecules.^120^
